# Bounds on the domination number and the metric dimension of co-normal product of graphs

**DOI:** 10.1186/s13660-018-1752-5

**Published:** 2018-07-04

**Authors:** Imran Javaid, Shahid ur Rehman, Muhammad Imran

**Affiliations:** 1 Centre for Advanced Studies in Pure and Applied Mathematics, Bahauddin Zakariya University, Multan, Pakistan; 2 Department of Mathematical Sciences, United Arab Emirates University, Al Ain, United Arab Emirates; 3 Department of Mathematics, School of Natural Sciences, National University of Sciences and Technology, Islamabad, Pakistan

**Keywords:** 05C12, 05C69, Dominating set, Resolving set, Adjacency resolving set, Co-normal product of graphs

## Abstract

In this paper, we establish bounds on the domination number and the metric dimension of the co-normal product graph $G_{H}$ of two simple graphs *G* and *H* in terms of parameters associated with *G* and *H*. We also give conditions on the graphs *G* and *H* for which the domination number of $G_{H}$ is 1, 2, and the domination number of *G*. Moreover, we give formulas for the metric dimension of the co-normal product $G_{H}$ of some families of graphs *G* and *H* as a function of associated parameters of *G* and *H*.

## Introduction

The domination number is a parameter that has appeared in numerous location problems [19] and in the analysis of social network problems [4]. The adjacency and non-adjacency relation between two vertices *u*, *v* in a graph *G* is denoted by $u\sim v$ and $u\nsim v$, respectively. A set $D\subseteq V(G)$, is a *dominating set* [22] of *G* if for every $v\in V(G)$, we have $v\in D$ or $v\sim u$ for some $u\in D$. The minimum cardinality of a dominating set in a graph *G* is called the *domination number* of *G*, denoted by $\gamma(G)$. The problem of finding a minimum size dominating set of a graph is in general NP-hard [13].

The metric dimension is a parameter that has appeared in robot navigation problems [20], strategies for the mastermind game [8], drug discovery problems [7, 17, 18], coin weighing problems [26], network discovery and verification problems [3]. The notation $d_{G}(u, v)$ or simply $d(u, v)$ denotes the distance between two vertices $u, v\in V(G)$, which is the length of a shortest path between them. For an ordered set $W=\{w_{1}, w_{2}, \ldots, w_{k}\}\subseteq V(G)$ and a vertex $v\in V(G)$, the *k*-vector $(d(v,w_{1}),d(v,w_{2}), \ldots,d(v,w_{k}))$, is called the *metric representation* of *v* with respect to *W*, denoted by $c_{W}(v)$. A set $W\subseteq V(G)$ is a *resolving (locating) set* [14, 27] of *G* if for any two distinct vertices $u, v\in V(G)$, $c_{W}(u)\neq c_{W}(v)$, which means that there exists at least one vertex $w\in W$ for which $d(v, w)\neq d(u, w)$. A minimum resolving set of *G* is called a *metric basis* of *G* and its cardinality is called the *metric dimension* of *G*, denoted by $\operatorname{dim}(G) (\operatorname{loc}(G))$. Gary and Johnson [13] noted that the problem of finding the metric dimension of a graph is NP-hard; however, its explicit construction is given by Khuller et al. [20]. The problem of finding the metric dimension of a graph is formulated as an integer programming problem by Chartrand et al. [7]. Relations between the domination number and the metric dimension of a graph are given in [1].

It is found in [2] that there are 256 possible products of any two graphs using the adjacency and the non-adjacency relations of these graphs. Several interesting types of graph products have been studied extensively in the literature. For instance, Caceres et al. [6], Yero et al. [29], Rodriguez-Velazquez et al. [24], Saputroa et al. [25], and Jannesari and Omoomi [16] investigated the metric dimension of the cartesian product, the corona product, the strong product, and the lexicographic product of graphs, respectively.

Out of product graphs, there is another well-known product graph introduced by Ore in 1962 [22], with the name *cartesian sum of graphs*. It was named *co-normal product of graphs* in [12]. Different properties and results regarding coloring and the chromatic number of the co-normal product of graphs are discussed in [5, 9, 11, 12, 23, 28]. In [21], Kuziak et al. studied the strong metric dimension of the co-normal product of graphs using the strong metric dimension of its components. In this paper, we have studied the domination number and the metric dimension of the co-normal product of graphs.

All considered graphs in this paper are non-trivial, simple and finite. In the next section, we describe some structural properties of the co-normal product of graphs. In Sect. 3, we study the domination number of the co-normal product of graphs and describe conditions on the graphs *G* and *H* so that the domination number of $G_{H}$ is 1, 2, and $\gamma(G)$. We also give bounds on the domination number of the co-normal product of graphs. In Sect. 4, we describe some properties of resolving sets in the co-normal product of graphs and give bounds on the metric dimension of the co-normal product of graphs. Moreover, we establish formulas for the metric dimension of some families of graphs.

## Methods

We use the combinatorial computing, combinatorial inequalities and graph theoretic analytic methods to prove the main results. The aim of this research is to provide bounds on the domination number and the metric dimension of the co-normal product of graphs and to give exact formulas for the metric dimension of some families of graphs.

### Co-normal product of graphs

**Figure 1:**
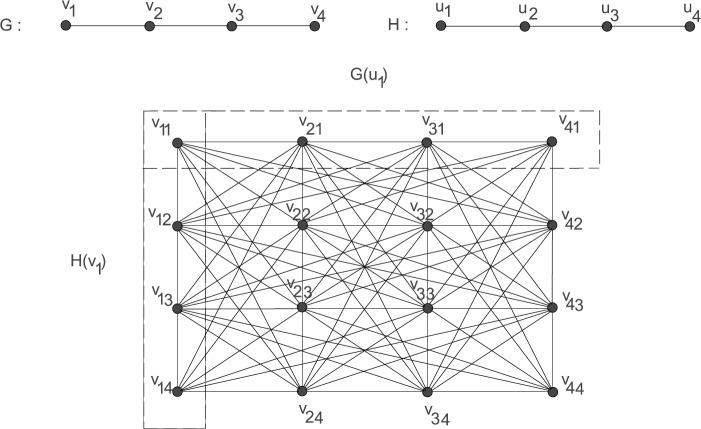
The co-normal product graph of $P_{4}$ and $P_{4}$

The *co-normal product* (the terminology we have adopted) of a graph *G* of order *m* with the vertex set $V(G)=\{v_{1},v_{2},\ldots ,v_{m}\}$ and a graph *H* of order *n* with the vertex set $V(H)=\{ u_{1},u_{2},\ldots,u_{n}\}$, is the graph $G_{H}$ with the vertex set $V(G)\times V(H)= \{v_{ij}=(v_{i},u_{j}) : v_{i}\in V(G) \text{ and } u_{j}\in V(H)\}$ and the adjacency relation defined as $v_{ij}\sim v_{rs}$ if $v_{i}\sim v_{r}$ in *G* or $u_{j}\sim u_{s}$ in *H*. All results given in this paper for $G_{H}$ also hold for $H_{G}$ due to the commutativity of this product. Figure 1 shows the co-normal product graph $G_{H}$ of two path graphs.

A graph having *n* vertices in which each vertex is adjacent to all other vertices is called a *complete graph*, denoted by $K_{n}$. In [12], Frelih and Miklavic discussed the connectivity of $G_{H}$ and proved the following theorem.

#### Theorem 1

(Frelih and Miklavic)

$G_{H}$ *is connected if and only if one of the following holds*:

1. $H= K_{n}$ *for some* $n\geq2$ *and* *G* *is connected*.
2. $G = K_{m}$ *for some* $m\geq2$ *and* *H* *is connected*.
3. *G* *and* *H* *are not null graphs and at least one of* *G* *or* *H* *is without isolated vertices*.

The diameter of a graph *G*, denoted by $\operatorname{diam}(G)$, is the maximum distance between any two vertices of *G*. If *G* is a disconnected graph then $\operatorname{diam}(G_{H})= \infty$. A graph having *n* vertices and no edges is called a *null graph*, denoted by $N_{n}$. In [21], Kuziak et al. discussed the diameter of $G_{H}$ and proved the following theorem.

#### Theorem 2

(Kuziak, Yero, Rodriguez-Velazquez)

*Let G and H be two non*-*trivial graphs such that at least one of them is non*-*complete and let* $n\geq2$ *be an integer*. *Then the following assertions hold*:

1. $\operatorname{diam}(G_{N_{n}})= \max\{2, \operatorname {diam}(G)\}$.
2. *G* *and* *H* *have isolated vertices*, *then* $\operatorname {diam}(G_{H})= \infty$.
3. *If neither* *G* *nor* *H* *has isolated vertices*, *then* $\operatorname{diam}(G_{H})=2$.
4. *If* $\operatorname{diam}(H)\leq2$, *then* $\operatorname{diam}(G_{H})=2$.
5. *If* $\operatorname{diam}(H) > 2$, *H* *has no isolated vertices and* *G* *is not a null graph having at least one isolated vertex*, *then* $\operatorname {diam}(G_{H}) = 3$.

The set of all vertices adjacent with a vertex $v\in V(G)$, is called the *open neighborhood* of *v* in *G*, denoted by $N_{G}(v)$ or simply $N(v)$. The cardinality of $N(v)$ is called the degree of *v* in *G*, denoted by $\operatorname{deg}_{G}(v)$ or simply $\operatorname{deg}(v)$. In the next two observations, we give formulas for the degree and the neighborhood of a vertex in $G_{H}$ using the structure of the co-normal product of graphs.

#### Observation 1

For any vertex $v_{ij}\in V(G_{H})$,

$$\operatorname{deg}(v_{ij})= \bigl\vert V(H) \bigr\vert \operatorname {deg}(v_{i})+\bigl( \bigl\vert V(G) \bigr\vert - \operatorname {deg}(v_{i})\bigr)\operatorname{deg}(u_{j}). $$

#### Observation 2

For any vertex $v_{ij}\in V(G_{H})$,

$$N(v_{ij})=N(v_{i})\times V(H)\cup\bigl(N(v_{i}) \bigr)^{c}\times N(u_{j}). $$

Two vertices having the same neighbors are called *false twins*. In the next theorem, we describe conditions for any two distinct vertices in $G_{H}$ to be false twins.

#### Theorem 3

*For any two distinct vertices* $v_{ij}$ *and* $v_{rs}$ *in* $G_{H}$, $N(v_{ij})= N(v_{rs})$ *if and only if* $N(v_{i})=N(v_{r})$ *in* *G* *and* $N(u_{j})=N(u_{s})$ *in* *H*.

#### Proof

Let $N(v_{ij})= N(v_{rs})$ in $G_{H}$, then, by Observation 2, we have $N(v_{i})\times V(H)\cup (N(v_{i})^{c}\times N(u_{j}))=N(v_{r})\times V(H)\cup(N(v_{r})^{c}\times N(u_{s}))$, which shows that $N(v_{i})=N(v_{r})$ in *G* and $N(u_{j})=N(u_{s})$ in *H*. The converse follows from the definition of the co-normal product of graphs. □

Let $v_{ij}\in V(G_{H})$, the set $C(v_{ij})=\{v_{kl}\in V(G_{H})| N(v_{kl})=N(v_{ij})\}$, is an equivalence class of false twins in $G_{H}$. Using Observation 2, we have the following straightforward lemma.

#### Lemma 1

*For any vertex* $v_{ij}\in V(G_{H})$, *we have* $C(v_{ij})= C(v_{i})\times C(u_{j})$, *where* $C(v_{i})$, $C(u_{j})$ *are equivalence classes of false twins in* *G* *and* *H*, *respectively*.

## Domination in co-normal product of graphs

A vertex of a graph *G* is a *dominating vertex* if its degree is $|V(G)|-1$. Throughout this section and the next section, the graphs *G*, *H* and $G_{H}$ are as described in Sect. 2. We define vertex sets, $G(u_{j})=\{v_{ij}: v_{i}\in V(G)\}\subseteq V(G_{H})$ and $H(v_{i})=\{v_{ij}: u_{j}\in V(H)\}\subseteq V(G_{H})$ for $v_{i}\in V(G)$ and $u_{j}\in V(H)$. In Fig. 1, we represent such classes. In the next two results, we give conditions on *G* and *H* for which $G_{H}$ have domination numbers 1 or 2.

### Lemma 2

*A vertex* $v_{ij}$ *is a dominating vertex in* $G_{H}$ *if and only if* $v_{i}$ *and* $u_{j}$ *are dominating vertices in* *G* *and* *H*, *respectively*.

### Proof

Let $v_{ij}$ be a dominating vertex in $G_{H}$. To show that $v_{i}$, $u_{j}$ are dominating in *G* and *H*, respectively, assume contrary that $v_{i}$ is not dominating in *G* so there exists $v_{k}\in V(G)$ such that $v_{k}\notin N(v_{i})$, then $v_{kj}\notin N(v_{ij})$ a contradiction.

Now suppose that $v_{i}$ and $u_{j}$ are dominating vertices in *G* and *H*, respectively, then, by Observation 1, we have $\operatorname{deg}(v_{ij})=|V(G)|\cdot|V(H)|-1$. □

### Lemma 3

*If* *G* *has a dominating vertex and* *H* *has no dominating vertex*, *then* $\gamma(G_{H})=2$.

### Proof

Suppose $v_{i}$ is a dominating vertex of *G*, so using the definition of co-normal product, $v_{ij}\sim v_{kl}$ for all $v_{kl}\in V(G_{H})$ with $v_{i}\neq v_{k}$. Also, *H* has no dominating vertex so there must be a vertex $u_{r}\in V(H)$ such that $u_{r} \notin N(u_{j})$, which shows that $v_{ir}\notin N(v_{ij})$. Now for any vertex $v_{k}\sim v_{i}$, the set $\{v_{ij}, v_{kl}\}$, is a dominating set for $G_{H}$ for any chosen vertex $v_{kl}\in H(v_{k})$. Hence, $\gamma(G_{H})=2$. □

A set $D\subseteq V(G)$ is a *total dominating set* [10] of *G*, if every vertex $v\in V(G)$ is adjacent to an element of *D*. The *total domination number*, denoted by $\gamma _{t}(G)$, is the cardinality of a minimum total dominating set for *G*. In the next theorem, we give conditions on *G* and *H* so that $\gamma(G_{H})=\gamma(G)$, by using the total domination number of *G*.

### Theorem 4

*For any two connected graphs* *G* *and* *H* *with* $2\leq\gamma(G)<\gamma(H)$, $\gamma(G_{H})=\gamma(G)$ *if and only if* $\gamma(G)=\gamma_{t}(G)$.

### Proof

Let $\gamma(G)=\gamma_{t}(G)$ and $D_{1}=\{\acute{v}_{1}, \acute {v}_{2}, \ldots, \acute{v}_{n_{1}}\}\subseteq V(G)$ be a minimum total dominating set of *G*. Consider the set $D=\{\acute{v}_{11}, \acute{v}_{22},\ldots, \acute{v}_{n_{1}n_{1}}\}\subseteq V(G_{H})$ where $\acute{v}_{ii}=(\acute{v}_{i}, \acute{u}_{i})$, $\acute{v}_{i}\in D_{1}$ and $\acute{u}_{i}\in V(H)$. To prove that $\gamma(G_{H})=\gamma (G)$, we only need to prove that *D* is a minimum dominating set for $G_{H}$. First, we show that *D* is a dominating for $G_{H}$. Clearly, $\acute{D}=\bigcup_{\acute{v}_{ii}\in D} N[\acute{v}_{ii}]\subseteq V(G_{H})$. Now for $v_{ij}\in V(G_{H})$ if $v_{ij}\in D$, then $v_{ij}\in \acute{D}$ and if $v_{ij}\notin D$ with $v_{i}\in D_{1}$, then there exists $\acute{v}_{k}\in D_{1}$ such that ${v}_{i}\sim\acute{v}_{k}$ because $D_{1}$ is a total dominating set of *G* so $v_{ij}\in\acute{D}$. Suppose $v_{i}\notin D_{1}$, then there exists $\acute{v}_{k}\in D_{1}$ such that $v_{i}\in N(\acute{v}_{k})$ so $v_{ij}\in\acute{D}$. Hence, *D* is dominating set for $G_{H}$.

Now to prove that *D* is a minimum dominating set, assume contrarily that $C\subseteq V(G_{H})$ be a minimum dominating set such that $|C|< \gamma (G)< \gamma(H)$. Consider the sets $D_{1}=\{v_{i}\in V(G)\mid v_{ij}\in C \text{ for some } u_{j}\in V(H)\}$ and $D_{2}=\{u_{j}\in V(H)\mid v_{ij}\in C \text{ for some } v_{i}\in V(G)\}$ then $D_{1}$ and $D_{2}$ are not dominating sets for *G* and *H*, respectively, which shows that there exists $v_{k}\in V(G)\setminus D_{1}$ and $u_{l}\in V(H)\setminus D_{2}$ such that $N(v_{k})\cap D_{1}=\emptyset$, $N(u_{l})\cap D_{2}=\emptyset$ and $N(v_{kl})\cap C= \emptyset$, a contradiction. Hence, *D* is a minimum dominating set for $G_{H}$.

Conversely, suppose $\gamma(G_{H})=\gamma(G)$ and *D* be a minimum dominating set for $G_{H}$. Let $D_{1}=\{v_{i}\in V(G)\mid v_{ij}\in D \text{ for some } u_{j}\in V(H)\}$ and $D_{2}=\{u_{j}\in V(H)\mid v_{ij}\in D\text{ for some }v_{i}\in V(G)\}$. Since $\gamma(G_{H})=\gamma(G)$, we have $|D_{1}|\leq\gamma(G)$ also $|D_{2}|< \gamma(H)$ by given condition. For $|D_{1}|< \gamma(G)$, there exist $v_{i}\in V(G)\setminus D_{1}$ and $u_{j}\in V(H)\setminus D_{2}$ such that $N(v_{i})\cap D_{1}=\emptyset$, $N(u_{j})\cap D_{2}=\emptyset$ and $N(v_{ij})\cap D=\emptyset$ and for $|D_{1}|= \gamma (G)$ with $D_{1}$ is not a dominating set for *G* a similar argument shows that *D* is not a dominating set for $G_{H}$. If $D_{1}$ is a minimum dominating set for *G*, we are to prove that $\gamma(G)=\gamma_{t}(G)$. Assume to the contrary that $\gamma(G)<\gamma_{t}(G)$, then there exist $v_{i}\in D_{1}$ such that $N(v_{i})\cap D_{1}=\emptyset$ and $u_{j}\in V(H)\setminus D_{2}$ such that $N(u_{j})\cap D_{2}=\emptyset$, which shows that $N(v_{ij})\cap D=\emptyset$, a contradiction to the assumption that $\gamma(G_{H})=\gamma(G)$. Hence, $\gamma(G)=\gamma_{t}(G)$. □

Lemma 2, shows that $\gamma(G_{H})=1$ if and only if $\gamma(G)=\gamma(H)=1$. In the next theorem, we give general bounds on the domination number of $G_{H}$.

### Theorem 5

*For any two connected graphs* *G* *and* *H*, $\min\{\gamma(G), \gamma(H)\}\leq\gamma(G_{H})\leq \gamma(G)\cdot\gamma(H)$.

### Proof

Let $D_{1}=\{\acute{v}_{1}, \acute{v}_{2}, \ldots, \acute {v}_{n_{1}}\}$, $D_{2}=\{\acute{u}_{1}, \acute{u}_{2}, \ldots, \acute{u}_{n_{2}}\}$ be dominating sets for *G*, *H*, respectively and $D=D_{1}\times D_{2}$. To show that *D* is a dominating set for $G_{H}$, consider a vertex $v_{ij}\in V(G_{H})$, we have following cases:

Case 1: If $v_{i}\in D_{1}$ and $u_{j}\in D_{2}$, then $v_{ij}\in \bigcup_{v_{ij}\in D}N[v_{ij}]$.

Case 2: If $v_{i}\in D_{1}$ and $u_{j}\notin D_{2}$, then there exists $u_{k}\in D_{2}$ such that $u_{j}\in N(u_{k})$. As $v_{ik}\in D$ so $v_{ij}\in N(v_{ik})$.

Case 3: If $v_{i}\notin D_{1}$ and $u_{j}\in D_{2}$, then there exists $v_{k}\in D_{1}$ such that $v_{i}\in N(v_{k})$. As $v_{kj}\in D$ so $v_{ij}\in N(v_{kj})$.

Case 4: Let $v_{i}\notin D_{1}$ and $u_{j}\notin D_{2}$, then there exist $v_{k}\in D_{1}$ and $u_{l}\in D_{2}$ such that $v_{i}\in N(v_{k})$ and $u_{j}\in N(u_{l})$ so $v_{ij}\in N(v_{kl})$ for $v_{kl}\in D$. Hence, *D* is a dominating set for $G_{H}$ and $\gamma(G_{H})\leq\gamma(G)\cdot\gamma(H)$.

Now for lower bound, consider $\gamma(G), \gamma(H)\geq1$. Suppose that $\gamma(G)=1$ and $\gamma(H)=1$, then, by Lemma 2, $\gamma(G_{H})=1$. Also for $\gamma(G)=1$ and $\gamma(H)\geq2$, Lemma 3, shows that $\gamma(G_{H})=2$. Suppose $2\leq\gamma(G)\leq\gamma (H)$ and $D\subset V(G_{H})$ be any set such that $|D|< \min\{\gamma(G), \gamma(H)\}$. To prove lower bound, we need to prove that *D* is not a dominating set for $G_{H}$. Let $D_{1}=\{v_{i}\in V(G)| v_{ij}\in D\text{ for some }u_{j}\in V(H)\}$ and $D_{2}=\{u_{j}\in V(H)| v_{ij}\in D\text{ for some }v_{i}\in V(G)\}$. Since $|D|< \min\{\gamma(G), \gamma(H)\}$, $D_{1}$ and $D_{2}$ are not dominating sets of *G* and *H*, respectively, which shows that there exist vertices $v_{k}\in V(G)\setminus D_{1}$ and $u_{l}\in V(H)\setminus D_{2}$ such that $N(v_{k})\cap D_{1}= \emptyset$ and $N(u_{l})\cap D_{2}= \emptyset$. Using the definition of the co-normal product of graphs $v_{ij}\in V(G_{H})\setminus D$ and $N(v_{ij})\cap D= \emptyset$. Hence, *D* is not a dominating set for $G_{H}$. □

Note that the lower bound given in Theorem 5, is attainable when $\gamma(G)=\gamma(H)$.

## Metric dimension in co-normal product of graphs

In this section, we study the properties of resolving sets in $G_{H}$ and establish formulas for the co-normal product of some families of graphs. In Theorem 10, we give bounds on the metric dimension of the co-normal product of a connected graph *G* and a graph *H* (not necessarily connected). In the rest of this paper, we assume *G* and *H* such that $G_{H}$ is connected. Moreover, $G_{H}$ has diameter at most two unless otherwise stated. In the next lemma, we will prove that, for every $v_{i}\in V(G)$, $u_{j}\in V(H)$ and an ordered set $W(v_{i})\subseteq H(v_{i})$, the distance of $v_{ij}, v_{kj}\in G(u_{j})$ to the vertices of $W(v_{i})$ is equal if $v_{ij}\notin W(v_{i})$ and $v_{i}\nsim v_{k}$ in *G*.

### Lemma 4

*Let* $G_{H}$ *has diameter* 2 *and* $W(v_{i})$ *be an ordered subset of* $H(v_{i})$ *for some* $v_{i}\in V(G)$. *If* $v_{ij}\notin W(v_{i})$ *for some* $u_{j}\in V(H)$, *then*, *for every* $v_{k}\nsim v_{i}$ *in* *G*, $c_{W(v_{i})}(v_{ij})=c_{W(v_{i})}(v_{kj})$.

### Proof

To show that $c_{W(v_{i})}(v_{ij})=c_{W(v_{i})}(v_{kj})$, we will show that $d(x, v_{ij})= d(x, v_{kj})$ for each $x\in W(v_{i})$. Let $x=v_{il}\in W(v_{i})$, for some $u_{l}\in V(H)$. Since $G_{H}$ has diameter 2, we have $d(x, v_{ij}), d(x, v_{kj})\in\{1, 2\}$. First suppose that $d(x, v_{ij})=1$, then $u_{j}\sim u_{l}$ in *H* and $v_{kj}\sim v_{il}$ in $G_{H}$. Hence, $d(x, v_{ij})= d(x, v_{kj})$. Now suppose that $d(x, v_{ij})=2$, which shows that $u_{l}\nsim u_{j}$ in *H* and $v_{kj}\nsim v_{il}$ in $G_{H}$. Hence, $d(x, v_{ij})= d(x, v_{kj})$. □

For $W\subseteq V(G_{H})$ and $W(v_{l})=W\cap H(v_{l})$; $v_{l}\in V(G)$, clearly $W=\bigcup_{v_{l}\in V(G)}W(v_{l})$ and $\{W(v_{l}); v_{l}\in V(G)\}$ gives a partition of *W*. For any vertex $v_{ij}\in V(G_{H})$, the code of $v_{ij}$ with respect to *W* can be represented as:

$$c_{W}(v_{ij})=\bigl(c_{W(v_{1})}(v_{ij}), c_{W(v_{2})}(v_{ij}),\ldots, c_{W(v_{m})}(v_{ij}) \bigr). $$

In the next lemma, we give conditions on an ordered set $W\subseteq V(G_{H})$ to be a resolving set for $G_{H}$.

### Lemma 5

*A set* $W\subseteq V(G_{H})$ *is a resolving set for* $G_{H}$ *if and only if for any two distinct vertices* $v_{ij},v_{rs}\in V(G_{H})$ *there exists at least one vertex* $v_{l}\in V(G)$ *such that* $N(v_{ij})\cap W(v_{l})\neq N(v_{rs})\cap W(v_{l})$, *where* $W(v_{l})=W\cap H(v_{l})$.

### Proof

Suppose *W* is a resolving set for $G_{H}$ and there exist two distinct vertices $v_{ij}$, $v_{rs}$ in $G_{H}$ such that, for every $v_{l}$ in *G*, we have $N(v_{ij})\cap W(v_{l})= N(v_{rs})\cap W(v_{l})$. Then $c_{W(v_{l})}(v_{ij})=c_{W(v_{l})}(v_{rs})$ for every $v_{l}\in V(G)$ because $G_{H}$ has diameter two and $c_{W}(v_{ij}) = c_{W}(v_{rs})$ because $c_{W}(v_{ij})=(c_{W(v_{1})}(v_{ij}), c_{W(v_{2})}(v_{ij}),\ldots, c_{W(v_{m})}(v_{ij}))$, a contradiction.

Conversely, suppose for any two distinct vertices $v_{ij},v_{rs}\in V(G_{H})$, there exists at least one vertex $v_{l}\in V(G)$ such that $N(v_{ij})\cap W(v_{l})\neq N(v_{rs})\cap W(v_{l})$. Since $G_{H}$ has diameter at most 2, we have $c_{W(v_{l})}(v_{ij})\neq c_{W(v_{l})}(v_{rs})$ and hence $c_{W}(v_{ij})\neq c_{W}(v_{rs})$ showing that *W* is a resolving set for $G_{H}$. □

In [15], the authors proved the following corollary, which gives the relation between resolving sets and false twins of a graph.

### Corollary 1

(Hernando, Mora, Pelaya, Seara, Wood)

*Suppose* *u*, *v* *are twins in a connected graph* *G* *and* *W* *resolves* *G*. *Then* *u* *or* *v* *is in* *W*. *Moreover*, *if* $u\in W$ *and* $v\notin W$, *then* $(W\setminus\{u\})\cup \{v\}$ *also resolves* *G*.

Using Corollary 1, and Lemma 1, if *H* has false twins then, for every resolving set *W* of $G_{H}$, $W\cap H(v_{i})\neq \emptyset$ for each $v_{i}\in V(G)$. In the next theorem, we give conditions on *G* and *H* for which there exists a resolving set *W* of $G_{H}$ such that $W\cap H(v_{i})= \emptyset$ for some $v_{i}\in V(G)$.

### Theorem 6

*Let* *G* *be a connected graph and* *H* *be an arbitrary graph such that* $\operatorname{diam}(G), \operatorname{diam}(H)\geq2$. *There exists a resolving set* *W* *for* $G_{H}$ *such that* $W\cap H(v_{i})= \emptyset$ *for some* $v_{i}\in V(G)$ *if and only if* *H* *has no false twins*.

### Proof

Let *W* be a resolving set of $G_{H}$ such that $W\cap H(v_{i})= \emptyset$, for some $v_{i}\in V(G)$. Assume contrary that $N(u_{j})= N(u_{s})$ for two distinct vertices $u_{j}, u_{s}\in V(H)$, then, by Lemma 3, $N(v_{kj})=N(v_{ks})$ in $G_{H}$ for each $v_{k}\in V(G)$ so $N(v_{ij})=N(v_{is})$ in $G_{H}$. As $W\cap H(v_{i})= \emptyset$ so by Corollary 1, *W* is not a resolving set for $G_{H}$, a contradiction.

Conversely, consider a set $W\subset V(G_{H})$ such that $V(G_{H})\setminus W= H(v_{i})$, for some $v_{i}\in V(G)$, where $v_{i}$ is not a dominating vertex in *G*. To prove the converse, we only need to prove that *W* is a resolving set for $G_{H}$. Let $v_{ij}, v_{il}\in H(v_{i})$ be two distinct vertices for some $u_{j}, u_{l}\in V(H)$. Since *H* have no false twins and diameter at least 2, there exists at least one vertex, say $u_{r}\in V(H)$, such that $u_{r}\in N(u_{j})$ or $u_{r}\in N(u_{l})$. Now for every $v_{k}\nsim v_{i}$ in *G*, we have $v_{kr}\in N(v_{ij})$ or $v_{kr}\in N(v_{il})$, which shows that $c_{W}(v_{ij})\neq c_{W}(v_{il})$. Hence, *W* is a resolving set for $G_{H}$. □

The following corollary directly follows from Theorem 6, which gives the relation between dominating sets and resolving sets of $G_{H}$, when both *G*, *H* are connected.

### Corollary 2

*For any two connected graphs* *G* *and* *H* *if at least one of* *G*, *H* *has false twins*, *then every resolving set of* $G_{H}$ *is a dominating set of* $G_{H}$.

In the next theorem, we give conditions on *G* and *H* for which the metric dimension of $G_{H}$ is the order of *G* times the metric dimension of *H*.

### Theorem 7

*Let* $C({u}_{1}),C({u}_{2}),\ldots, C({u}_{k})$ *be the distinct equivalence classes of false twins in a connected graph* *H* *with the property that* $|C({u}_{i})|\neq1$ *for each* $1\leq i\leq k$ *and* *G* *be a connected graph having no false twins*, *then* $\operatorname {dim}(G_{H})=|V(G)|\cdot \operatorname{dim}(H)$.

### Proof

Since $N(v_{i})\neq N(v_{k})$, for any two distinct vertices $v_{i}, v_{k}\in V(G)$, *G* has $|V(G)|$ distinct equivalence classes of false twins. Lemma 1, shows that the co-normal product $G_{H}$ has $|V(G)|\cdot k$ equivalence classes of false twins such that no class has cardinality 1, so $\operatorname{dim}(G_{H})=\sum _{i=1}^{|V(G)|}\sum _{j=1}^{k}|C(v_{ij})| - |V(G)|\cdot k$. Also $|C(v_{ij})| =|C({u}_{j})|$ for each $v_{i}\in V(G)$ and ${u}_{j}\in\{{u}_{1}, {u}_{2}, \ldots, {u}_{k}\}$, which shows that $\operatorname{dim}(G_{H})=\sum _{i=1}^{|V(G)|}\sum _{j=1}^{k}| C({u}_{j})| - |V(G)|\cdot k$. Hence, $\operatorname {dim}(G_{H})=|V(G)|\cdot\operatorname{dim}(H)$. □

Let $P_{m}$; $m\geq4$ be a path graph and $K_{n_{1}, n_{2},\ldots, n_{k}}$; $n_{i}\geq2$ for each *i*, be a complete multipartite graph have *k* distinct equivalence classes of false twins. Since $P_{m}$ have no false twins, by Theorem 7, we have the following corollary.

### Corollary 3

*If* $G=P_{m}$; $m\geq4$ *and* $H= K_{n_{1}, n_{2},\ldots, n_{k}}$, *then* $\operatorname{dim}(G_{H})= m\prod _{j=1}^{j=k}(n_{j}-1)$.

In [16], Jannesari and Omoomi introduced the concept of the adjacency metric dimension of a graph and used it to find the metric dimension of lexicographic product of graphs. A function $a: V(G)\times V(G)\rightarrow\{0,1,2\}$ defined as:

$$a(u,v) = \textstyle\begin{cases} 0 &\mbox{if } u = v,\\ 1 &\mbox{if } u \sim v,\\ 2 &\mbox{if } u \nsim v . \end{cases} $$

for $u,v\in V(G)$, is called the *adjacency function* of *G*. The *k*-vector $(a(v,w_{1}), a(v,w_{2}), \ldots, a(v,w_{k}))$ for a vertex $v\in V(G)$, is called the *adjacency metric representation* of *v* with respect to *W*, denoted by $c^{a}_{W}(v)$. A set *W* is an *adjacency resolving set* for *G* if for any two distinct vertices $u, v\in V(G)$, $c^{a}_{W}(u)\neq c^{a}_{W}(v)$ or $N(u)\cap W\neq N(v)\cap W$. A minimum adjacency resolving set of *G* is called an *adjacency basis* of *G* and its cardinality is called the *adjacency metric dimension* of *G*, denoted by $\operatorname {adim}(G)$. They also gave that if *G* is a connected graph with diameter 2, then $\operatorname{dim}(G) = \operatorname{adim}(G)$ but the converse is not true because $\operatorname{dim}(C_{6}) = 2 = \operatorname{adim}(C_{6})$, while $\operatorname{diam}(C_{6}) = 3$. Our next lemma directly follows from the definition of adjacency basis and the fact that the induced subgraph $\langle H(v_{i}) \rangle$ of $G_{H}$ is isomorphic to *H*, for each $v_{i}\in V(G)$.

### Lemma 6

*If* $G_{H}$ *has diameter at most* 3 *and* $W_{2}$ *is an adjacency basis for* *H*, *then*, *for any* $v_{i}\in V(G)$, *the vertices of* $H(v_{i})$ *are resolved by its subset* $W_{2}(v_{i})=\{v_{i}\}\times W_{2}$.

**Figure 2:**
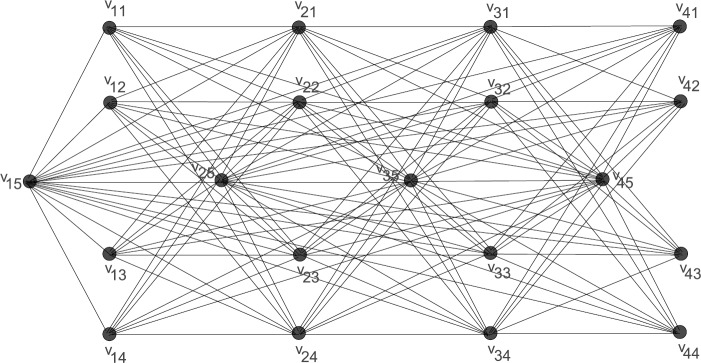
The co-normal product graph of $P_{4}$ and $S_{4}$

Now consider a path graph $P_{4}$ having the vertex set $V(P_{4})=\{v_{1}, v_{2}, v_{3}, v_{4}\}$ such that $v_{i}\sim v_{i+1}$; $i\leq3$ and a star graph $S_{4}$ having the vertex set $V(S_{4})=\{u_{1}, u_{2}, u_{3}, u_{4}, u_{5}\}$ such that $u_{5}\sim u_{i}$; $1\leq i\leq4$. The co-normal product graph of $P_{4}$ and $S_{4}$ is shown in Fig. 2. Note that, for every adjacency basis $W_{2}$ of $S_{4}$, $c^{a}_{W_{2}}(u_{5})=(1, 1, 1)$ and $W=\bigcup_{v_{i}\in V(P_{4})}(\{v_{i}\}\times W_{2})$ is not a resolving set for $G_{H}$. Let **1** represents a vector whose each entry is 1 and **2** represents a vector whose each entry is 2, i.e. $\boldsymbol {1}=(1,1,\ldots, 1)$ and $\boldsymbol{2}=(2,2,\ldots,2)$. In the next theorem, we provide conditions under which $W=\bigcup_{v_{i}\in V(G)} W_{2}(v_{i})$ is a resolving set for $G_{H}$, where $W_{2}$ is an adjacency basis of *H* and $W_{2}(v_{i})=\{v_{i}\}\times W_{2}$.

### Theorem 8

*Let* *G* *be a connected graph having no false twins and* *H* *be a graph such that* $G_{H}$ *has diameter at most three*. *If there exists an adjacency basis* $W_{2}$ *of* *H* *such that* $c^{a}_{W_{2}}(u_{j})\neq\boldsymbol{1}$ *for all* $u_{j}\in V(H)$, *then* $\operatorname{dim}(G_{H})\leq|V(G)|\cdot\operatorname{adim}(H)$.

### Proof

Let $W_{2}(v_{i})=\{v_{i}\}\times W_{2}$ and $W=\bigcup_{v_{i}\in V(G)} W_{2}(v_{i})$ or $W= V(G)\times W_{2}$. By Lemma 6, $W_{2}(v_{i})$ resolves all the vertices of $H(v_{i})$. To show that *W* is a resolving set for $G_{H}$, consider two distinct vertices $v_{ij}, v_{kl}\in V(G_{H})\setminus W$ such that $v_{i}\neq v_{k}$. Since *G* has no false twins, we have $N(v_{i})\neq N(v_{k})$ for all $v_{i}\neq v_{k}\in V(G)$ and $N(v_{ij})\cap W\neq N(v_{kl})\cap W$ for $u_{j}=u_{l}$, *W* resolves $v_{ij}$, $v_{kl}$. Now for $u_{j}\neq u_{l}$, we have $N(u_{j})\cap W_{2}\neq N(u_{l})\cap W_{2}$ also $N(v_{i})\cap V(G)\neq N(v_{k})\cap V(G)$, which shows that *W* resolves $v_{ij}$, $v_{kl}$. Hence, by Lemma 5, $W=\bigcup_{v_{i}\in V(G)} W_{2}(v_{i})$ is a resolving set for $G_{H}$. □

### Corollary 4

*Let* *G* *be a complete graph and* $H\neq K_{n}$ *be an arbitrary graph*. *If* *H* *has an adjacency basis* $W_{2}$ *such that* $c^{a}_{W_{2}}(u_{j})\neq\boldsymbol{1}$ *for all* $u_{j}\in V(H)$, *then* $\operatorname{dim}(G_{H})= |V(G)|\cdot\operatorname{adim}(H)$.

### Proof

Since *G* is complete, *G* has no false twins. Also, $W_{2}$ satisfies the condition of Theorem 8, so $\operatorname {dim}(G_{H})\leq |V(G)|\cdot\operatorname{adim}(H)$. Now for some $u_{j}\in W_{2}$, consider $W=V(G)\times(W_{2}\setminus\{u_{j}\})$ and note that, for any $v_{i}\in V(G)$, $W(v_{i})=\{v_{i}\}\times(W_{2}\setminus\{u_{j}\})$ will not resolves the vertices of $H(v_{i})$, because $W_{2}$ is an adjacency basis of *H*, so there exists $u_{l}\in V(H)\backslash W_{2}$ such that $c^{a}_{W_{2}\setminus\{u_{j}\}}(u_{l})=c^{a}_{W_{2}\setminus\{u_{j}\}}(u_{j})$ in *H*, which shows that $c_{W(v_{k})}(v_{il})=c_{W(v_{k})}(v_{ij})$ for all $v_{k}\neq v_{i}$ because *G* is complete. Hence, $\operatorname {dim}(G_{H})\geq |V(G)|\cdot\operatorname{adim}(H)$. □

In the next theorem, we give a formula for the metric dimension of $G_{H}$ when *G* is complete and *H* is a graph for which each adjacency basis $W_{2}$ has one vertex $u_{j}\in V(H)\setminus W_{2}$ such that $c^{a}_{W_{2}}(u_{j})= \boldsymbol{1}$.

### Theorem 9

*Let* *G* *be a complete graph and* $H\neq K_{n}$ *be an arbitrary graph*. *If for each adjacency basis* $W_{2}$ *of* *H*, *there exists a vertex* $u_{j}\in V(H)\setminus W_{2}$ *such that* $c^{a}_{W_{2}}(u_{j})= \boldsymbol{1}$, *then* $\operatorname{dim}(G_{H})= |V(G)|\cdot(\operatorname{adim}(H)+ 1)-1$.

### Proof

By using Lemma 6, $W(v_{i})=\{v_{i}\}\times W_{2}$ will resolve the vertices of $H(v_{i})$. Since *G* is complete, $c_{W(v_{k})}(v_{ij})= \boldsymbol{1}$ for all $v_{k}\neq v_{i}$. Also $c_{W(v_{i})}(v_{ij})= \boldsymbol{1}$ for each $v_{i}\in V(G)$. Hence, $W=\bigcup_{v_{i}\in V(G)} W(v_{i})$ is not a resolving set for $G_{H}$. Also the induced subgraph of the vertex set $G(u_{j})=\{v_{ij}|v_{i}\in V(G)\}$ is isomorphic to *G* and *G* is complete. Hence, $\operatorname {dim}(G_{H})= |V(G)|\cdot\operatorname{adim}(H)+ |V(G)|-1$. □

Since $G_{H}$ is complete if and only if *G* and *H* are complete, $\operatorname{dim}(G_{H})=|V(G)|\cdot|V(H)|-1$. Also, $G_{H}\cong H$ if *G* is trivial and $G_{H}\cong G$ if *H* is trivial. Note that $\operatorname{dim}(G_{H})= \operatorname{adim}(G)\cdot\operatorname {adim}(H)$ if and only if one of *G* or *H* is trivial. In the next theorem, we give bounds for the metric dimension of $G_{H}$ when *G* and *H* are non-trivial and at least one is not a complete graph.

### Theorem 10

*Let* *G* *be a connected graph and* $H\neq K_{n}$ *be an arbitrary graph*, *then*

$$\operatorname{adim}(H)\cdot\operatorname{adim}(G)< \operatorname {dim}(G_{H})\leq \bigl\vert V(G) \bigr\vert \cdot\operatorname {adim}(H)+ \bigl\vert V(H) \bigr\vert \cdot\operatorname{adim}(G). $$

### Proof

Let $W= W_{1}\times V(H)\cup V(G)\times W_{2}$, where $W_{1}$, $W_{2}$ are adjacency basis of *G* and *H* respectively. Let $W(v_{i})=W\cap H(v_{i})$ for $v_{i}\in V(G)$ and $W(u_{j})=W\cap G(u_{j})$ for $u_{j}\in V(H)$. For any vertex $v_{ij}\in V(G_{H})$, the metric representation is of the form $c_{W}(v_{ij})=(c_{W(v_{1})}(v_{ij}), c_{W(v_{2})}(v_{ij}),\ldots, c_{W(v_{m})}(v_{ij}))$ or $c_{W}(v_{ij})=(c_{W(u_{1})}(v_{ij}), c_{W(u_{2})}(v_{ij}),\ldots, c_{W(u_{n})}(v_{ij}))$. For any two distinct vertices $v_{ij}, v_{kl}\in V(G_{H})\setminus W$, we have $v_{i}, v_{k}\notin W_{1}$ and $u_{j}, u_{l}\notin W_{2}$. To prove that *W* is a resolving set for $G_{H}$, we discuss the following cases:

Case 1: Let $v_{i}=v_{k}$ and $W_{2}(v_{i})=\{v_{i}\}\times W_{2}\subseteq W(v_{i})$. Lemma 6, shows that $W_{2}(v_{i})$ resolves the vertices of $H(v_{i})$ also $W_{2}(v_{i})\subseteq W(v_{i})$ shows that $c_{W(v_{i})}(v_{ij})\neq c_{W(v_{i})}(v_{kl})$. Hence, $c_{W}(v_{ij})\neq c_{W}(v_{kl})$.

Case 2: Let $u_{j}=u_{l}$ and $W_{1}(u_{j})=W_{1}\times \{u_{j}\}\subseteq W(u_{j})$. $W_{1}(u_{j})$ resolves the vertices of $G(u_{j})$, which shows that $c_{W(u_{j})}(v_{ij})\neq c_{W(u_{j})}(v_{kl})$. Hence, $c_{W}(v_{ij})\neq c_{W}(v_{kl})$.

Case 3: Let $v_{i}\neq v_{k}$ and $u_{j}\neq u_{l}$. Since $W_{1}$ and $W_{2}$ are adjacency bases for *G* and *H*, respectively, we have $N(v_{i})\cap W_{1}\neq N(v_{k})\cap W_{1}$ and $N(u_{j})\cap W_{2}\neq N(u_{l})\cap W_{2}$. Also, $W= W_{1}\times V(H)\cup V(G)\times W_{2}$ shows that $N(v_{ij})\cap W\neq N(v_{kl})\cap W$, which implies *W* is a resolving set for $G_{H}$.

For the lower bound, let $W_{1}$ and $W_{2}$ be adjacency basis for *G* and *H*, respectively and $W=W_{1}\times W_{2}$. We consider the following cases:

Case 1: Suppose *G* or *H* has false twins. Since, for every $v_{i}\in V(G)\setminus W_{1}$, we can have $W\cap H(v_{i})=\emptyset$, by Theorem 6, *W* is not a resolving set for $G_{H}$ if *H* has false twins. A similar argument holds if *G* has false twins.

Case 2: Suppose neither *G* nor *H* have false twins. As $W_{2}$ is an adjacency basis for *H* so there exists at least one vertex $u_{j}\in W_{2}$ such that $c_{W_{2}\setminus\{u_{j}\}}(u_{j})=c_{W_{2}\setminus\{u_{j}\} }(u_{l})$ for some $u_{l}\in V(H)\setminus W_{2}$. Also, $W\cap H(v_{i})=\emptyset$ for $v_{i}\in V(G)\setminus W_{1}$ and the definition of the co-normal product graph gives $c_{W}(v_{ij})= c_{W}(v_{il})$. Hence, *W* is not a resolving set for $G_{H}$. □

For a complete graph *G* and a null graph *H*, Theorem 2(1) shows that $\operatorname{diam}(G_{H})=2$ and the metric dimension of $G_{H}$ is given in the next theorem.

### Theorem 11

*If* *G* *is a complete graph and* *H* *is a null graph*, *then* $\operatorname{dim}(G_{H})=|V(G)|\cdot(|V(H)|-1)$.

### Proof

Let $V(G)=\{v_{1}, v_{2}, \ldots, v_{m}\}$ and $V(H)=\{u_{1}, u_{2}, \ldots, u_{n}\}$. It is clear from the definition of co-normal product that, for each $v_{i}$, $N(v_{ij})=N(v_{ik})$ for all $1\leq j, k\leq n$. So any resolving set must contain at least $n-1$ vertices from each $H(v_{i})$, which shows that $\operatorname {dim}(G_{H})\geq m(n-1)$. Since *H* is a null graph, we have $c_{H(v_{i})\setminus \{v_{ij}\}}(v_{ij})= \boldsymbol{2}$ for each *i* and $c_{H(v_{k})}(v_{ij})= \boldsymbol{1}$ for each $k\neq i$, which shows that any subset of $V(G_{H})$ containing $n-1$ vertices from each $H(v_{i})$ will be a resolving set for $G_{H}$. Hence, $\operatorname{dim}(G_{H})=m(n-1)$. □

In the next theorem, we give formula for the metric dimension of $G_{H}$ when *G* is a path graph and *H* is a star graph.

### Theorem 12

*For any two integers* $m, n\geq2$, *if* *G* *is a path graph and* *H* *is a star graph having order* *m* *and* $n+1$ *respectively*, *then* $\operatorname{dim}(G_{H})= m\cdot\operatorname {dim}(H)+\operatorname{adim}(G)$.

### Proof

Let $V(G)=\{v_{1}, v_{2}, \ldots, v_{m}\}$ and $V(H)=\{u_{0}, u_{1}, u_{2}, \ldots, u_{n}\}$, where $\operatorname {deg}(u_{0})=n$ in *H*. Also, $N(u_{k})=N(u_{l})$ for all $1\leq k, l\leq n$, by using Lemma 3, we have $N(v_{ik})=N(v_{il})$ for each *i*. So, any resolving set *W* for *G* must contain at least $n-1$ vertices from each $H(v_{i})$. Since $\operatorname{deg}(u_{0})=n$, by the definition of a co-normal product $d(v_{i0}, v_{ij})=1$ for all $1\leq i\leq m$ and $1\leq j\leq n$, which means that the vertices of $G(u_{0})$ are not resolved by any of $v_{ij}$, $1\leq i\leq m$, $1\leq j\leq n$. Also, $d(v_{i0}, v_{j0})\leq2$ in $G_{H}$ and induced subgraph of $G(u_{0})$ is isomorphic to *G* so we must choose $\operatorname{adim}(G)$ vertices from $G(u_{0})$, which shows that $\operatorname{dim}(G_{H})=m\cdot\operatorname {dim}(H)+ \operatorname{adim}(G)$. □

## Conclusions

To study the product graphs with respect to graph theoretic parameters is always an important problem. In this paper, we have studied two parameters, the domination number and the metric dimension of the co-normal product of two graphs *G* and *H*. These two parameters have a lot of applications in networks and facility location problems. We have given conditions on *G* and *H* under which the graph $G_{H}$ has the domination number 1, 2 and $\gamma(G)$. We also proved that, for any two connected graphs *G* and *H*, $\min\{\gamma(G), \gamma(H)\}\leq \gamma(G_{H})\leq \gamma(G)\gamma(H)$. We described some properties of resolving sets of $G_{H}$ and gave conditions on *G* and *H* such that $\operatorname {dim}(G_{H})= |V(G)|\cdot\operatorname{dim}(H)$. We have also given conditions on *G* and *H* under which $\operatorname{dim}(G_{H})\leq |V(G)|\cdot\operatorname{adim}(H)$. For a complete graph *G* and a non-complete graph *H*, we have given conditions on *H* under which $\operatorname{dim}(G_{H})= |V(G)|\cdot\operatorname{adim}(H)$ and $\operatorname{dim}(G_{H})= |V(G)|\cdot(\operatorname{adim}(H)+ 1)-1$. For a connected graph *G* and a non-complete graph *H*, we proved that $\operatorname{adim}(H)\cdot\operatorname {adim}(G)<\operatorname{dim}(G_{H})\leq|V(G)|\cdot\operatorname {adim}(H)+ |V(H)|\cdot\operatorname{adim}(G)$. We have also given explicit formulas for the metric dimension of the co-normal product of a path graph and a complete multipartite graph, a complete graph and a null graph, a path graph and a star graph for the first time. Our derived inequality relations can be very helpful in the characterizations of graphs with given metric dimension or given domination number.
